# Characterization of primary aroma compounds in Pu‐erh raw tea sourced from various regions using gas chromatography‐mass spectrometry and headspace solid‐phase microextraction

**DOI:** 10.1111/1750-3841.17562

**Published:** 2024-12-01

**Authors:** Lijuan Du, Yanping Ye, Jinliang Shao, Yutong Wang, Guolei Zhu, Hua Jiang, Hongcheng Liu, Zhenhuan Liu

**Affiliations:** ^1^ Institute of Quality Standards & Testing Technique Yunnan Academy of Agricultural Science Kunming China; ^2^ Faculty of Food Science and Technology Yunnan Agricultural University Kunming China; ^3^ Key Laboratory of State Forestry and Grassland Adiministration on Highly‐Efficient Utilization of Forestry Biomass Resources in Southwest China Southwest Forestry University Kunming China; ^4^ Yunnan Huaying Technology Company Limited Kunming China

**Keywords:** enantiomers, KEGG pathway, Pu‐erh raw tea, volatiles

## Abstract

The assessment of aroma plays a crucial role in determining the characteristics and quality of Pu‐erh tea. The volatile compounds present in Pu‐erh raw tea sourced from five regions in Yunnan Province have been studied using gas chromatography‐mass spectrometry (GC‐MS) in conjunction with headspace solid phase microextraction (HS‐SPME). A total of 69 aroma‐active compounds were identified, with alcohols, ketones, and aldehydes being the predominant component types. Furthermore, notable variations were observed among four pairs of volatile terpenoid enantiomers. Significant discrimination was achieved using orthogonal partial least squares discrimination analysis (*R*(*x* ^2^) = 0.926, *R*(*y* ^2^) = 0.843, *Q* ^2 ^= 0.501). Moreover, 33 important differential compounds were identified based on variable importance in the projection values (VIP > 1.0) and one‐way ANOVA analysis (*p* < 0.05). Subsequently, a KEGG pathway analysis was conducted, revealing enrichment pathways primarily focused on the mevalonate pathway, steroid biosynthesis, and fatty acid biosynthesis. Ultimately, the key volatile compounds and major aroma‐contributing compounds were successfully identified.

## 1 INTRODUCTION

Tea is widely recognized as a popular beverage globally, valued for its superior flavor and numerous health benefits (Shao et al., 2022; Zhai et al., 2022). With a rich historical significance, tea is highly esteemed by a diverse range of consumers for its distinct aroma, taste, and medicinal properties. The main classifications of Chinese tea encompass green tea, yellow tea, white tea, oolong tea, black tea, dark tea, and Pu‐erh tea (Guo et al., 2023; B. Y. Wang et al., 2022; Wong et al., 2022). Pu‐erh tea, primarily cultivated in the Yunnan province of China, is recognized as a geographical indication product of Yunnan, with its main ingredient being sun‐dried green tea from the Yunnan large leaf species within the designated protection area. The production process of Pu‐erh raw tea encompasses steps such as withering, rolling, sun drying, steaming, and pressing. After high‐temperature steam treatment and compression, a Pu erh raw tea cake is obtained, which has a compact appearance and is suitable for long‐term storage. (Guo et al., 2023; Li et al., 2024). Renowned for its distinct postfermented nature, Pu‐erh tea has gained popularity due to its perceived health benefits and unique aroma profile.

Flavor is one of the most critical criteria to evaluate the quality of tea. The flavor quality is influenced by factors, such as the tea plant cultivar, geographical positions, harvest conditions, processing and storage conditions. For example, Wuyi rock tea is known for its unique “rocky aroma and floral fragrance,” which is cultivated in the Wuyi Mountain area of Fujian (Zhai et al., 2022). Qingzhuan tea, famous as “aged fragrance”, is manufactured mainly in Chibi City, Hubei province (P. Liu et al., 2022). Qimen red tea is renowned for its “Qimen fragrance”, mainly produced in the adjacent Guichi (Yun, 2020). Additionally, Pu‐erh raw tea exhibits diverse aroma characteristics based on varying planting conditions (elevation, precipitation, air temperatures, etc.). For example, tea from Iceland has a distinct honey aroma, while tea from Xigui has a strong aroma of orchids, and the aroma characteristics of Pu‐erh raw tea produced in Xishuangbanna and Lincang are also diverse (Y. Q. Xu et al., 2016). Certainly, the diverse aromas and fragrances found in different types of teas are indicative of the unique flavor profiles inherent in each tea species. The composition of tea aromas is influenced by a multitude of factors that collectively contribute to the distinct aroma components and flavor substances present in teas. The “stale” aroma of Pu‐erh tea is primarily associated with the broad class of volatile methoxybenzene compounds, involving 1,2,3‐trimethoxybenzene, 1,2,3‐trimethoxy‐5‐methylbenzene, and 4‐ethyl‐1,2‐dimethoxybenzene, etc.

While fresh tea leaves contain varying amounts of astringent and bitter compounds, scarcely emit aroma, subsequently, a wide array of flavorful compounds are formed through processing. Tea can be classified into unfermented green tea, semi‐fermented oolong tea, and fully fermented black tea based on the degree of fermentation during processing. According to the Food and Agriculture Organization of the United Nations statistics database (https://www.fao.org/faostat/), the yearly world production is above 4.4 million tons (2022), with China (33%) and India (13%) being the top two tea producers in the world. The fermentation process is essential for the development of the distinctive aromas and flavors found in Pu‐erh raw tea, primarily due to microbial activity. The extracellular enzymes produced by microorganisms play a significant role in the oxidation, condensation, and degradation of endogenous compounds in Pu‐erh tea, thereby influencing its color, aroma, and flavor profile (B. Y. Wang et al., 2022; Wong et al., 2022). The predominant aroma categories in Pu‐erh tea are floral (38.62%) and fruity (16.55%) (Y. Q. Xu et al., 2016). Moreover, alcohols like geraniol and linalool contribute to the floral notes. Ketones, the second most abundant group of odorants in tea, contribute to floral, fruity, and woody aroma characteristics. Aldehydes have been identified as a contributing factor to the citrus‐like and green flavor of tea infusion and potentially impart bitter‐almond‐like, honey‐like, and fatty odor notes. (Guo et al., 2023; Zhai et al., 2022)

Notably, these volatile compounds owing stereocentric structures can result in the detection of optical mixtures with varying enantiomeric ratios (ERs). It has been demonstrated that the distribution of chiral volatiles is greatly influenced by factors such as cultivar, geographical origin, leaf area, storage duration, and processing techniques (Shao et al., 2022). Various enantiomers may exhibit distinct properties, including differences in odor characteristics, odor thresholds, and potential biological activities (Zhu et al., 2017). For instance, R‐linalool (odor threshold of 0.8 µg/L) has a woody and lavender‐like smell, S‐linalool (odor threshold of 7.4 µg/L) has a sweet, flowery, and orange‐like aroma, and racemic linalool (odor threshold of 6 µg/L) has a floral and fruit‐like odor. Z‐linalool oxide (furanoid) has a floral and wood‐like aroma, and E‐linalool oxide (furanoid) (odor threshold of 0.32 µg/L) has a fruit and grass‐like aroma. R‐limonene (odor threshold of 34 µg/L) has a fruit‐like aroma and S‐limonene has a fat‐like aroma and racemic limonene (odor threshold of 13 µg/L) has a citrus and carrot‐like odor. Z‐2‐heptenal (odor threshold of 40 µg/L) has a fat and mushroom‐like aroma, and E‐2‐heptenal (odor threshold of 13–18 µg/L) has a fat and fruit‐like aroma (odor threshold of 53 ng/L). S‐α‐ionone (odor threshold of 20–40 µg/L) has a woody odor, whereas R‐α‐ionone (odor threshold of 0.5–5 µg/L) has a violet‐like aroma, and racemic α‐ionone (odor threshold of 0.4–1.0 µg/L) has a strong sweet‐floral odor. (J. Y. Chen et al., 2022; Jin et al., 2023; P. P. Liu et al., 2023; L. Ma et al., 2022; B. Y. Wang et al., 2022; Wei et al., 2024; Wu et al., 2023; Zeng et al., 2023; Zhai et al., 2022). Given the variability in aroma profiles, it is hypothesized that differing enantiomeric compositions may elicit diverse fragrance attributes, thereby significantly impacting the overall aroma quality of tea (Zhu et al., 2017).

This study successfully established a simple and effective analytical approach for enantiomeric and quantitative analysis of volatiles in Pu‐erh raw tea, utilizing HS‐SPME combined with chiral GC‐MS technology, to further investigate the influence of origin and environment on aroma quality and identify key odorants. The multivariate statistical analysis sheds light on the distribution characteristics of volatiles and their enantiomers in various sources of Pu‐erh raw tea, while also identifying the key volatile compounds and major aroma‐contributing compounds. The findings contribute to the development and enhancement of the origin tracing system for Pu‐erh raw tea in Yunnan Province, China.

## 2 MATERIALS AND METHODS

### 2.1 Sample collection and preparation

**FIGURE 1:**
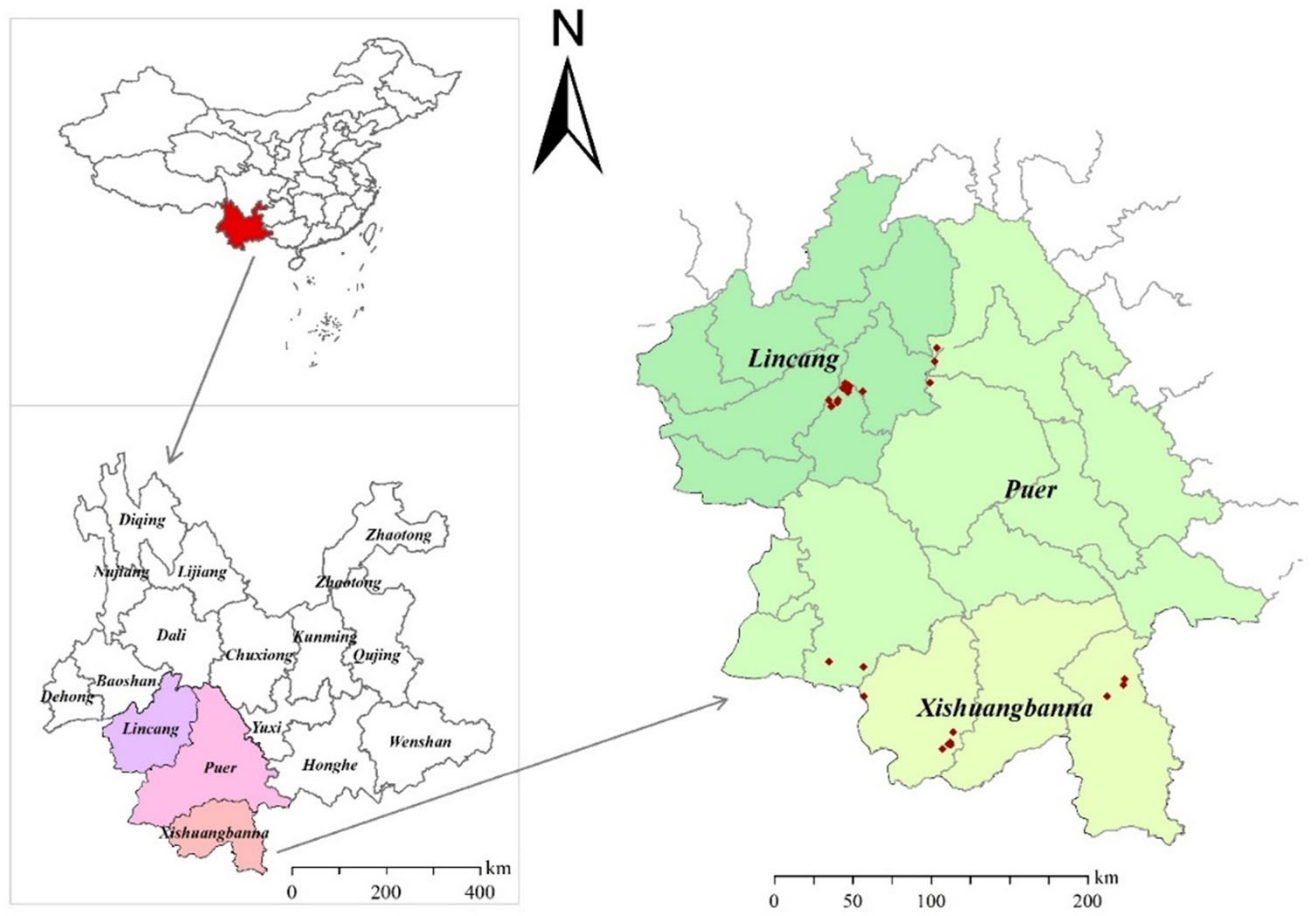
Distribution of Pu‐erh raw tea origin places.

The Pu‐erh raw tea materials were collected from Lincang, Puer, and Xishuangbanna city or prefecture in Yunnan Province in 2021 (Figure 1), and were made from fresh tea leaves mainly through fixing, rolling, and drying. After that, the same material was allocated equivalently and was further produced into Pu‐erh raw tea at local factories according to their traditional postfermentation methods. All raw materials for Pu‐erh raw tea were ancient tree teas from a single origin place, which was no situation of multiple origin combinations (Table 1).

**TABLE 1:** Information of Pu‐erh raw tea samples.

Sampling location		No.	Soil texture
Shuangjiang Autonomous County, Lincang City (SJ)	Bingdao Laozhai	Tea‐01, Tea‐02	Sandy loam
	Dijie Laozhai	Tea‐03, Tea‐04	Sandy loam
	Nanpo Laozhai	Tea‐05, Tea‐06	Sandy loam
	Nuowu	Tea‐07, Tea‐08	Sandy loam
	Bawai Laozhai	Tea‐09, Tea‐10	Sandy loam
	Xiaohuzhai	Tea‐11, Tea‐12	Sandy loam
	Baka	Tea‐13, Tea‐14	Sandy loam
	Dahuzhai	Tea‐15, Tea‐16	Loam
Linxiang District, Lincang City (LX)	Xigui Manglu Mountain	Tea‐17, Tea‐18, Tea‐19	Sandy loam
Lancang AutonomousCounty, Puer City (LC)	Wenji Jingmai Mountain	Tea‐20, Tea‐21, Tea‐22	Loam
Menghai County, Xishuangbanna Prefecture (MH)	Menghai Laobanzhang	Tea‐23	Loam
	Menghai Hekai	Tea‐24, Tea‐25	Loam
	Banzhang	Tea‐26, Tea‐27	Sandy loam
Mengla County, Xishuangbanna Prefecture (ML)	Yiwu Laomansa	Tea‐28, Tea‐29, Tea‐30	Sandy loam

### 2.2 Chemicals and reagents

HPLC‐grade acetonitrile, isopropanol, and methanol were purchased from Merck. Ethyl caprate (as an internal standard) and normal alkane (C7‐C40, 1000 µg/mL) standard substances were bought from Aladdin Biochemical Technology Co., LTD.

### 2.3 Volatile extraction by HS‐SPME

The volatiles from Pu‐erh raw tea were extracted by the headspace solid‐phase microextraction (HS‐SPME) method (Fan, 2022). Before use, the extraction fiber heads need to be aged for 1 h at 250°C in the GC‐MS injection port. 0.200 g tea powder was placed into a 20 mL headspace bottle, sealed immediately after adding 10 µL ethyl caprate (12.7 µg/mL) as an interior label and 2 mL boiling water, extracted for 40 min at 80°C, took out the fiber head right away, and inserted into the injection port for dissociative absorption (5 min, 240°C) and GC‐MS analysis.

### 2.4 Qualitative and quantitative analyses of volatiles

A Thermo Fisher TSQ 9610 GC system, coupled with a TSQ 9610 mass spectrometer (Thermo Fisher) was used to identify volatile compounds in all samples. A Thermo Fisher TG‐WAXMS capillary column (50 m × 0.3 mm × 0.25 µm) was employed to separate the volatiles. Helium (>99.999 %) was used as the carrier gas at a constant flow rate of 1 mL/min with unsplit stream sampling. The GC oven temperature was held at 50°C, ramped at a rate of 10°C/min to 150°C for 30 min, and then ramped to 240°C with the same rate. The mass spectrometer conditions were as follows: interface temperature of 250°C, ion source temperature of 230°C, mass scan range of 45–540 m/z, and solvent delay time of 3.0 min.

Identification of volatile compounds was based on information on mass spectra from the national standard technical spectrum database (NIST17.L) combined with AMDIS deconvolution, and furtherly identified by retention indices (RIs, with the computational Equation 1). RIs were calculated after analyzing the n‐alkane series (C7‐C40) under the same chromatographic conditions, and then the RIs of each compound between the experimental and reported values were compared after peak alignment and impurities removal.

(1) $$RI=\frac{100n+100\left(t_{i}-t_{n}\right)}{t_{n}+1-t_{n}}\;,$$

where *t_i_* is the retention time of the target compound (min) and *n* and *n*+1 are the number of n‐alkane carbon before and after the outflow of the target compound.

Subsequently, the internal standard method was used to relatively quantify the finally determined aroma compounds, and the ratio between the peak area of tested compounds and the total peak area of internal standard was utilized in the relative contents of the corresponding compounds. The relative content of each volatile was calculated with Equation (2).

(2) $$\begin{array}{ccc} & & \mathrm{Relative}\;\mathrm{content}\;=\\ & & \frac{\mathrm{Individual}\;\mathrm{compound}\;\mathrm{content}}{\mathrm{Total}\;\mathrm{content}}\;\times 100\%, \end{array}$$

### 2.5 Statistical analyses

The data are calculated by dried weight (*n *= 3) and the graphics processing is analyzed by ORIGIN and SIMCA in this paper. Data are analyzed by ORIGIN, whose homogeneity of variance and normality are evaluated by the Bartlett test. Data with uniform variance are analyzed by one‐way analysis of variance (ANOVA), conducting Holm–Bonferroni multiple comparisons, and the difference significance test (*p* < 0.05). To further analyze the possible signaling pathways affecting differential metabolites, the differential metabolites were imported into the MetaboAnalyst (https://www.metaboanalyst.ca) online website to analyze the main enriched KEGG biosynthetic pathways.

## 3 RESULTS AND DISCUSSION

### 3.1 Volatile profiling analysis of Pu‐erh raw tea

**FIGURE 2:**
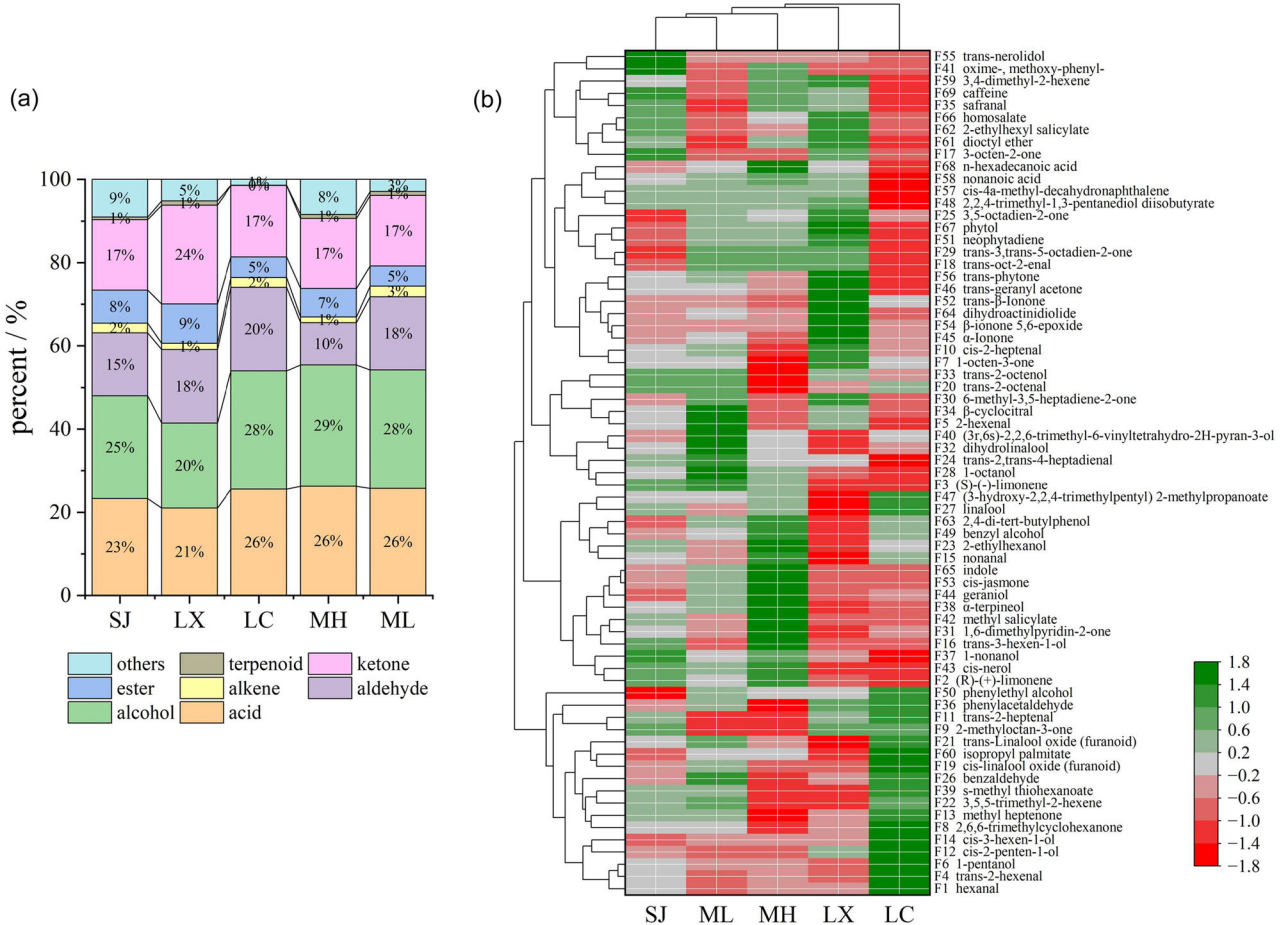
Volatiles in Pu‐erh raw tea from different regions. (a) percent in each kind of volatiles. (b) Heatmap analyses.

The volatiles of Pu‐erh raw tea are very complex and found at trace levels. Up to now, over 700 volatiles have been identified in tea; however, only a part of volatile substances elicit typical odor notes, leading to the variability of tea solely in terms of aroma (Feng et al., 2020). In this study, there are 69 volatiles identified from Pu‐erh raw tea, including alcohols, aldehydes, ketones, esters, acids, terpenes, alkenes, and others. Every location of Pu‐erh raw tea has its own aroma characteristics that are generally obviously different from each other in volatiles. Specifically, the number of volatiles identified in SJ, LX, LC, MH, and ML are 69, 61, 51, 63, and 59, respectively, among which 55 are their common volatiles. (Table 2; Figure 2)

**TABLE 2:** Volatile components of raw tea from different regions.

No.	Name	Formula	Matching degree	RI	Retention time/min	Average contents/%					Oder characteristics
						SJ	LX	LC	MH	ML	
F1	Hexanal	C_6_H_12_O	90.5	1083	5.736	0.89 ± 0.39^b^	0.47 ± 0.41^b^	1.88 ± 0.51^a^	0.58 ± 0.58^b^	0.25 ± 0.43^b^	Fruity/grass
F2	(R)‐(+)‐limonene	C_10_H_16_	90.9	1199	8.033	0.21 ± 0.28^a^	0.07 ± 0.12^a^	ND	0.24 ± 0.23^a^	0.14 ± 0.24^a^	Fruity
F3	(S)‐(−)‐limonene	C_10_H_16_	85.3	1191	8.251	0.15 ± 0.27^a^	ND	ND	0.11 ± 0.24^a^	0.20 ± 0.34^a^	Fatty
F4	Trans‐2‐hexenal	C_6_H_10_O	91.7	1216	8.929	0.83 ± 0.69^b^	0.24 ± 0.42^b^	1.83 ± 0.29^a^	0.46 ± 0.46^b^	0.35 ± 0.60^b^	Fruity/floral/fatty
F5	2‐Hexenal	C_6_H_10_O	92.0	1213	8.932	0.36 ± 0.60^ab^	0.55 ± 0.48^ab^	ND	0.18 ± 0.41^ab^	0.91 ± 0.84^a^	Grass/fatty
F6	1‐pentanol	C_5_H_12_O	95.7	1250	9.673	0.43 ± 0.23^ab^	0.19 ± 0.18^b^	0.74 ± 0.18^a^	0.31 ± 0.41^b^	0.28 ± 0.26^b^	Fruity/fatty
F7	1‐Octen‐3‐one	C_8_H_16_O	93.8	978	10.991	0.49 ± 0.35^ab^	0.97 ± 0.26^a^	0.49 ± 0.43^ab^	ND	0.52 ± 0.53^ab^	Mushroom/earthy
F8	2,2,6‐Trimethylcyclohexanone	C_9_H_16_O	94.0	1317	11.37	0.44 ± 0.21^ab^	0.38 ± 0.07^ab^	0.69 ± 0.08^a^	0.28 ± 0.26^b^	0.47 ± 0.09^ab^	Fruity
F9	2‐Methyl‐3‐octanone	C_9_H_18_O	83.2	1323	11.383	0.39 ± 0.48^a^	0.36 ± 0.63^a^	0.37 ± 0.64^a^	ND	ND	
F10	Cis‐2‐heptenal	C_7_H_12_O	80.1	1322	11.457	0.49 ± 0.54^ab^	0.92 ± 0.8^a^	0.42 ± 0.37^ab^	0.09 ± 0.19^b^	0.66 ± 0.42^ab^	Fatty/mushroom
F11	Trans‐2‐heptenal	C_7_H_12_O	88.4	1323	11.621	0.24 ± 0.40^a^	0.22 ± 0.38^a^	0.32 ± 0.55^a^	ND	ND	Fatty/fruity/grass
F12	Cis‐2‐penten‐1‐ol	C_5_H_10_O	87.4	1318	11.725	0.1 ± 0.18^b^	0.28 ± 0.26^ab^	0.53 ± 0.47^a^	ND	ND	Fruity/rubber
F13	6‐Methyl‐5‐hepten‐2‐one	C_8_H_12_O	89.1	1339	11.967	1.14 ± 0.14^ab^	1.01 ± 0.15^b^	1.22 ± 0.05^a^	0.80 ± 0.08^c^	1.11 ± 0.11^ab^	
F14	Cis‐3‐hexen‐1‐ol	C_6_H_12_O	93.3	1382	13.110	0.45 ± 0.33^b^	0.61 ± 0.12^b^	1.30 ± 0.14^a^	0.55 ± 0.66^b^	0.55 ± 0.09^b^	Floral
F15	Nonanal	C_9_H_18_O	94.5	1391	13.402	0.51 ± 0.36^ab^	0.15 ± 0.26^b^	0.61 ± 0.15^ab^	0.74 ± 0.33^a^	0.40 ± 0.12^ab^	Fruity/floral
F16	Trans‐3‐hexen‐1‐ol	C_6_H_12_O	88.7	1367	13.428	0.17 ± 0.27^a^	ND	ND	0.26 ± 0.36^a^	ND	Grass
F17	3‐Octen‐2‐one	C_8_H_12_O	85.8	1411	13.617	0.12 ± 0.19^a^	0.10 ± 0.17^a^	ND	ND	ND	Nutty
F18	Trans‐2‐octenal	C_8_H_16_O	91.5	1429	14.308	0.22 ± 0.08^a^	0.31 ± 0.05^a^	0.20 ± 0.01^a^	0.31 ± 0.61^a^	0.31 ± 0.03^a^	Fatty/nutty
F19	Cis‐linalool oxide (furanoid)	C_10_H_18_O_2_	93.4	1444	14.559	0.68 ± 0.33^c^	0.42 ± 0.15^c^	1.65 ± 0.14^a^	0.55 ± 0.32^c^	1.15 ± 0.19^b^	Woody/floral
F20	1‐Octen‐3‐ol	C_8_H_16_O	92.2	1450	14.807	8.21 ± 2.09^a^	6.33 ± 1.34^ab^	7.57 ± 0.69^a^	4.69 ± 1.71^b^	8.60 ± 0.91^a^	Grass/mushroom
F21	Trans‐linalool oxide (furanoid)	C_10_H_18_O_2_	89.3	1452	15.280	2.08 ± 0.85^ab^	0.89 ± 0.32^b^	3.01 ± 2.61^a^	1.80 ± 0.71^ab^	2.73 ± 0.48^a^	Fruity/floral/grass/earthy
F22	3,5,5‐Trimethyl‐2‐hexene	C_9_H_18_	83.3	1489	15.693	1.62 ± 0.92^ab^	0.51 ± 0.88^b^	1.75 ± 0.12^ab^	0.52 ± 0.50^b^	1.92 ± 0.20^a^	
F23	2‐Ethyl‐1‐hexanol	C_8_H_18_O	94.1	1491	15.740	1.34 ± 0.63^a^	0.89 ± 0.12^a^	1.22 ± 0.16^a^	1.61 ± 0.29^a^	1.08 ± 0.17^a^	Fruity/floral/fatty
F24	Trans‐2, trans‐4‐heptadienal	C_7_H_10_O	87.5	1495	15.927	1.96 ± 0.83^ab^	1.42 ± 0.59^b^	ND	1.43 ± 0.88^b^	2.82 ± 0.11^a^	Floral/fatty/nutty
F25	3,5‐Octadien‐2‐one	C_8_H_12_O	89.4	1522	16.561	0.92 ± 0.71^a^	1.65 ± 0.14^a^	1.21 ± 0.12^a^	1.24 ± 0.09^a^	1.43 ± 0.06^a^	Fruity/fatty/mushroom
F26	Benzaldehyde	C_7_H_6_O	96.8	1520	16.655	2.84 ± 0.40^b^	2.76 ± 0.24^b^	3.53 ± 0.37^a^	2.45 ± 0.43^b^	3.57 ± 0.29^a^	Fruity/nutty
F27	Linalool	C_10_H_18_O	92.3	1547	17.225	6.01 ± 1.27^a^	3.31 ± 0.45^b^	6.75 ± 0.39^a^	6.03 ± 1.58^a^	4.91 ± 0.18^ab^	Fruity/floral
F28	1‐Octanol	C_8_H_18_O	95.2	1557	17.420	0.64 ± 0.11^a^	0.61 ± 0.05^a^	0.59 ± 0.07^a^	0.66 ± 0.06^a^	0.71 ± 0.09^a^	Fruity/fatty
F29	Trans‐3, trans‐5‐octadien‐2‐one	C_8_H_12_O	89.4	1570	17.775	0.71 ± 0.43^a^	0.81 ± 0.09^a^	0.71 ± 0.05^a^	0.80 ± 0.10^a^	0.80 ± 0.03^a^	Floral
F30	6‐Methyl‐3,5‐heptadiene‐2‐one	C_8_H_12_O	89.9	1602	18.309	0.72 ± 0.23^a^	0.80 ± 0.12^a^	0.70 ± 0.08^a^	0.69 ± 0.07^a^	0.79 ± 0.03^a^	
F31	1,6‐Dimethylpyridin‐2‐one	C_7_H_9_NO	82.0	1614	18.698	0.55 ± 0.70^a^	ND	0.25 ± 0.44^a^	1.23 ± 1.69^a^	0.37 ± 0.64^a^	
F32	Dihydro‐linalool	C_10_H_16_O	89.6	1613	18.734	2.29 ± 1.40^ab^	1.35 ± 0.20^b^	1.99 ± 1.73^ab^	2.39 ± 0.63^ab^	3.61 ± 0.17^a^	Fruity/floral/woody
F33	Trans‐2‐octen‐1‐ol	C_8_H_16_O	91.9	1618	18.818	1.68 ± 0.36^a^	1.52 ± 0.20^a^	1.32 ± 0.15^ab^	0.96 ± 0.10^b^	1.72 ± 0.10^a^	Fatty/rubber/chemical
F34	β‐Cyclocitral	C_10_H_16_O	89.3	1611	18.929	0.38 ± 0.16^a^	0.43 ± 0.03^a^	0.33 ± 0.29^a^	0.32 ± 0.19^a^	0.51 ± 0.08^a^	Fruity/Grass
F35	Safranal	C_10_H_14_O	83.2	1616	19.405	0.18 ± 0.24^a^	0.16 ± 0.28^a^	ND	0.20 ± 0.28^a^	ND	Grass
F36	Phenylacetaldehyde	C_8_H_8_O	94.8	1641	19.519	5.14 ± 1.51^bc^	8.1 ± 1.75^a^	9.22 ± 0.39^a^	2.77 ± 2.78^c^	7.07 ± 0.49^ab^	Floral/honey
F37	1‐Nonanol	C_9_H_20_O	91.1	1660	19.818	0.08 ± 0.07^a^	0.04 ± 0.07^a^	ND	0.07 ± 0.10^a^	0.05 ± 0.09^a^	Fatty/floral/chemical
F38	α‐Terpineol	C_10_H_18_O	94.9	1697	20.693	1.43 ± 0.73^ab^	0.84 ± 0.06^b^	1.13 ± 0.09^b^	2.21 ± 0.86^a^	1.61 ± 0.05^ab^	Floral/grass/woody
F39	s‐Methyl thiohexanoate	C_7_H_14_OS	88.2	1412	20.934	0.39 ± 0.25^a^	ND	0.55 ± 0.05^a^	ND	0.33 ± 0.29^a^	Fruity/chemical
F40	(3r,6s)‐2,2,6‐Trimethyl‐6‐vinyltetrahydro‐2H‐pyran‐3‐ol	C_10_H_18_O_2_	84.1	1739	22.165	0.63 ± 0.38^bc^	0.17 ± 0.29^c^	0.77 ± 0.16^b^	0.87 ± 0.23^b^	1.49 ± 0.03^a^	Floral
F41	Oxime‐, methoxy‐phenyl‐	C_8_H_9_NO_2_	83.7	1767	22.202	3.21 ± 4.21^a^	ND	ND	2.17 ± 3.08^a^	ND	
F42	Methyl salicylate	C_8_H_8_O_3_	93.7	1765	22.467	1.68 ± 0.53^a^	1.27 ± 0.08^a^	1.31 ± 0.2^a^	2.32 ± 1.5^a^	1.36 ± 0.04^a^	Nutty/grass
F43	Cis‐nerol	C_10_H_18_O	82.8	1797	22.973	0.23 ± 0.35^a^	ND	ND	0.28 ± 0.28^a^	0.19 ± 0.17^a^	Floral
F44	Geraniol	C_10_H_18_O	89.9	1847	23.976	1.20 ± 0.79^b^	1.03 ± 0.11^b^	1.33 ± 0.1^b^	2.60 ± 0.52^a^	1.80 ± 0.04^ab^	Fruity/floral
F45	α‐Ionone	C_13_H_20_O	86.8	1840	24.047	1.64 ± 0.61^a^	2.18 ± 0.11^a^	1.62 ± 0.15^a^	1.56 ± 0.49^a^	1.69 ± 0.22^a^	Floral/grass
F46	Trans‐geranyl acetone	C_13_H_22_O	91.0	1859	24.154	1.63 ± 0.37^a^	1.92 ± 0.19^a^	1.42 ± 0.05^a^	1.58 ± 0.3^a^	1.63 ± 0.28^a^	Grass
F47	(3‐Hydroxy‐2,2,4‐trimethyl pentyl) 2‐methylpropanoate	C_12_H_24_O_3_	93.2	1872	24.439	0.75 ± 0.23^a^	0.66 ± 0.37^a^	0.82 ± 0.25^a^	0.79 ± 0.21^a^	0.77 ± 0.29^a^	
F48	2,2,4‐Trimethyl‐1,3‐pentanediol diisobutyrate	C_16_H_30_O_4_	85.1	1879	24.587	0.39 ± 0.40^a^	0.52 ± 0.45^a^	ND	0.39 ± 0.36^a^	0.39 ± 0.67^a^	
F49	Benzyl alcohol	C_7_H_8_O	95.5	1870	24.633	0.43 ± 0.06^bc^	0.38 ± 0.06^c^	0.51 ± 0.07^b^	0.66 ± 0.05^a^	0.47 ± 0.04^bc^	Fruity/floral/nutty
F50	Phenylethyl alcohol	C_8_H_10_O	95.2	1907	25.341	0.37 ± 0.27^b^	0.64 ± 0.10a^b^	0.84 ± 0.12^a^	0.67 ± 0.41^ab^	0.71 ± 0.05^ab^	Floral/honey
F51	Neophytadiene	C_20_H_38_	88.0	1922	25.589	0.23 ± 0.17^bc^	0.79 ± 0.23^a^	0.03 ± 0.05^c^	0.49 ± 0.36^ab^	0.59 ± 0.03^a^	
F52	Trans‐β‐ionone	C_13_H_20_O	95.2	1941	25.858	4.04 ± 0.88^a^	3.03 ± 0.21^a^	4.23 ± 0.24^a^	3.90 ± 1.08^a^	4.02 ± 0.62^a^	Floral/woody
F53	Cis‐jasmone	C_11_H_16_O	88.3	1961	25.945	0.08 ± 0.11^bc^	ND	ND	0.42 ± 0.07^a^	0.18 ± 0^b^	Fruity
F54	β‐ionone 5,6‐epoxide	C_13_H_20_O_2_	89.5	1962	26.944	1.91 ± 0.56^b^	4.48 ± 2.73^a^	1.78 ± 0.19^b^	1.72 ± 0.46^b^	1.72 ± 0.01^b^	Fruity/woody
F55	Trans‐nerolidol	C_15_H_26_O	82.4	2042	27.870	0.75 ± 2.68^a^	0.14 ± 0.02^a^	0.04 ± 0.08^a^	0.21 ± 0.03^a^	0.19 ± 0.04^a^	Floral/fatty/woody
F56	Trans‐phytone	C_18_H_36_O	89.5	2131	29.526	0.89 ± 0.25^b^	1.48 ± 0.24^a^	0.49 ± 0.09^c^	0.84 ± 0.31^bc^	1.11 ± 0.35^ab^	Fatty
F57	Cis‐4a‐methyl‐decahydronaphthalene	C_11_H_20_	82.0	2157	30.066	1.06 ± 0.68^a^	1.01 ± 0.87^a^	ND	0.97 ± 0.89^a^	0.96 ± 0.83^a^	
F58	Nonanoic acid	C_9_H_18_O_2_	90.5	2170	30.331	0.39 ± 0.15^a^	0.46 ± 0.1^a^	ND	0.61 ± 0.23^a^	0.46 ± 0.09^a^	Fatty
F59	3,4‐Dimethyl‐2‐hexene	C_8_H_16_	88.4	2189	30.636	0.57 ± 0.32^a^	0.81 ± 0.05^a^	0.36 ± 0.32^a^	0.73 ± 0.46^a^	0.44 ± 0.38^a^	
F60	Isopropyl palmitate	C_19_H_38_O_2_	90.2	2235	31.609	0.13 ± 0.14^b^	0.04 ± 0.08^b^	0.64 ± 0.52^a^	0.29 ± 0.33^b^	0.33 ± 0.12^ab^	Fatty
F61	Dioctyl ether	C_16_H_34_O	87.7	2279	32.270	0.79 ± 1.14^a^	1.12 ± 0.76^a^	ND	0.71 ± 1.08^a^	ND	
F62	2‐Ethylhexyl salicylate	C_15_H_22_O_3_	96.8	2301	32.669	1.78 ± 2.5^a^	2.55 ± 1.45^a^	ND	0.66 ± 1.19^a^	ND	
F63	2,4‐di‐tert‐Butylphenol	C_14_H_22_O	94.7	2321	32.994	12.39 ± 4.86^a^	11.15 ± 2.76^a^	14.91 ± 4.24^a^	16.99 ± 8.01^a^	14.45 ± 3.01^a^	
F64	Dihydro‐actinidiolide	C_11_H_16_O_2_	93.5	2327	33.457	1.21 ± 0.33^b^	2.08 ± 0.32^a^	1.06 ± 0.20^b^	1.19 ± 0.20^b^	1.35 ± 0.27^b^	Floral
F65	Indole	C_8_H_7_N	90.2	2445	35.241	0.1 ± 0.21^b^	ND	ND	0.75 ± 0.69^a^	0.31 ± 0.27^ab^	Floral
F66	homosalate	C_16_H_22_O_3_	91.9	2454	35.254	0.97 ± 1.45^a^	1.24 ± 0.87^a^	ND	0.47 ± 0.92^a^	ND	
F67	Phytol	C_20_H_40_O	92.5	2622	37.847	1.48 ± 0.72^c^	4.73 ± 0.42^a^	0.78 ± 0.26^c^	2.92 ± 1.75^b^	2.97 ± 0.26^b^	Floral
F68	Hexadecanoic acid	C_16_H_32_O_2_	92.2	2931	42.347	0.53 ± 0.26^bc^	0.69 ± 0.12^b^	0.13 ± 0.11^c^	1.18 ± 0.65^a^	0.56 ± 0.06^bc^	Fatty
F69	Caffeine	C_8_H_10_N_4_O_2_	92.4	3147	47.327	3.2 ± 3.59^a^	2.48 ± 0.92^a^	1.25 ± 0.35^a^	2.96 ± 2.45^a^	1.14 ± 1.55^a^	Floral

*Notes*: Multiple comparisons are performed using the Holm–Bonferroni method; data in the same row followed by a different lowercase letter indicates a significant difference between groups (*p* < 0.05). F1‐F69 shows the code for the volatiles; ND shows not detected; matching degree shows the conformity with the indicational results from the national standard technical spectrum database (NIST17.L) combined with AMDIS deconvolution, the higher the value, the better.

*Source*: C. Chen et al. (2022); J. Y. Chen et al. (2022); Difford et al. (2022); Du et al. (2024); Duflos et al. (2010); Feng et al. (2020); Ficke et al. (2021); Globisch et al. (2014); Gou et al. (2023); Guo et al. (2023); Hiller et al. (2019); Jin et al. (2023); Joll et al. 2010); P. Liu et al. (2022); P. P. Liu et al. (2023); L. Ma et al. (2022); L. L. Ma et al. (2023); Nie et al. (2020); Pang et al. (2019); Shi et al. (2019); Wakai et al. (2019); B. Y. Wang et al. (2022); C. Wang et al. (2016); Wei et al. (2024); Wu et al. (2023); Yang et al. (2016); Yun (2020); Zeng et al. (2023); Zhao et al. (2020) (www.vcf‐online.nl/VcfHome.cfm).

There exists a certain difference of volatiles among these five origin places—mainly including 2,4‐di‐tert‐butylphenol, linalool, dihydro‐linalool, linalool oxide (furanoid), α‐ionone, trans‐β‐ionone, β‐ionone 5,6‐epoxide, phenylacetaldehyde, benzaldehyde, phytol, α‐terpineol, dihydro‐actinidiolide, geraniol, nonanal, cis‐3‐hexen‐1‐ol, indole, and so on (Table 2). The majority of these compounds reported in Pu‐erh raw tea contribute to the major aroma components (such as floral, fruity, woody, honey, fatty, and even some unpleasant odor, etc.), playing a significant role in aroma quality forming (L. L. Ma et al., 2023; S. S. Xu et al., 2021). The heat map was performed to visually reveal the differences between the volatiles in the 5 origin places, where green represents the higher content of given compounds and red represents the lower content. The results showed obvious differences among the five sites of Pu‐erh raw tea (Figure 2b).

Compared with some previous reports, the numbers of identified volatile compounds using the HS‐SPME method are much higher than those identified by other extraction methods, such as solid‐phase microextraction (SPME) and steam distillation extraction (W. Ma et al., 2021). As shown in Figure 2a, there is an obvious difference in volatiles among five productions, among which the main volatiles are alcohols, acids, aldehydes, and ketones. Specifically, the proportion of alcohols in the aroma components of Pu'er raw tea in Menghai County is the highest at 29%, while in Linxiang District, it is the lowest at 20%; the proportion of acids is highest at 26% in Menghai County, while Linxiang District has the lowest proportion at 21%; the proportion of aldehydes in Lincang County is the highest at 20%, while in Menghai County, it is the lowest at 10%; the proportion of ketones in Linxiang District is the highest at 24%, while in other areas are all at 17%.

Alcohols account for the largest ratio of total volatiles in Pu‐erh raw tea. They typically have floral and fruity aromas, which are observed to make up around 50% of the total volatiles in Chinese congou black teas (Zhai et al., 2022). The cell permeability would enhance when the water loss of fresh leaves happens, resulting in the combination of β‐ glucosidase and glycosides to release alcohol with floral and fruity flavors (D. M. Wang et al., 2001). Specifically, two monoterpene alcohols geraniol (fruity and floral) and linalool (fruity, floral, and woody) appear most frequently and are the key odorants having a flowery odor note and relatively low odor thresholds (0.6 and 3.2 µg/L in water, respectively), so they play a crucial role in the formation of the aroma of Pu‐erh raw tea (Li et al., 2021; Nie et al., 2020; Qiu et al., 2024; Zhai et al., 2022). There is a different tendency in five origin places of these two volatiles. The relative content of linalool in Pu‐erh raw tea derived from LC possesses the highest (6.75%), the next from MH (6.03%) and SJ (6.01%), and the last from LX (3.31%). The relative content of geraniol from LX is also the lowest (1.03%), while the highest is from MH (2.60%). Twenty alcohols are aroma‐active compounds in tea and make up the highest proportion of odorants in Figure 2a. Among them, the relative content of linalool and its oxides are higher than others, reflecting the relatively high levels of geranyl pyrophosphate, making up 33.0%‐53.4% of the total (highest in LC and lowest in LX), the precursor for geraniol or linalool (Zhai et al., 2022).

Fifteen aroma‐active aldehydes were found, contributing flowery, grass, woody, mushroom‐like, and fruity odor notes (Table 2). β‐Ionone derived from carotenoid during the fermentation or the heating‐drying manufacturing process is a representative ketone of the aroma‐active compounds in tea, making it the main contributor to floral aroma (Silva, 2014; Zhai et al., 2022). β‐Ionone 5,6‐epoxide including a methyl‐ketone functional group is the key intermediate of the above degradation (Joll et al., 2010; Silva, 2014). The relative content of β‐ionone from LX (4.93%) is highest of 5 locations, and lowest from ML and MH (4.02% and 3.90%). There is also the highest relative content of β‐ionone 5,6‐epoxide in Pu‐erh raw tea derived from LX (4.48%), and the lowest from MH and ML (both are 1.72%).

In addition to alcohols and ketones, aldehydes are the third largest group of volatiles detected in Pu‐erh raw tea. In absolute numbers, aldehydes are the second largest group of odorants. According to the related literature, aldehydes contribute to the fruity and floral of tea infusion, but some are responsible for grass, honey, fatty, nutty, mushroom, and even rubber odor (Gou et al., 2023; Guo et al., 2023; P. Liu et al., 2022; P. P. Liu et al., 2023; Lv et al., 2014; Pang et al., 2019; W. Ma et al., 2021). Aldehydes in tea are known to be formed by two main formation pathways: lipoxygenase‐mediated lipid oxidation and Strecker degradation (Zhai et al., 2022). As present in Table 2, 12 aldehydes with odor descriptions were reported in previous research. Hexanal, trans‐2‐hexenal, 2‐hexenal, cis‐2‐heptenal, trans‐2‐heptenal, and trans, trans‐2, 4‐heptadienal are regarded as aroma substances originating from the lipoxygenase‐mediated oxidation of linolenic acid and oleic acid, which are major unsaturated fatty acids in tea (C. Chen et al., 2022; Du et al., 2024; Wakai et al., 2019). Strecker aldehydes including phenylacetaldehyde and benzaldehyde are generally derived from the reaction of amino acid and dimethyl compound under heat treatment, the reduction of which is focused on the enzyme inactivation, evaporation, or degradation/rearrangement reactions for corresponding alcohols (Shi et al., 2019; Wei et al., 2024; Zhai et al., 2022). Specifically, aldehydes originating from the first pathway account for 26.4–36.9% of the total (highest in SJ and lowest in LC), while aldehydes coming from the second make about 59.9–72.0% of the total (highest in LC and lowest in MH).

Only four acids, 2, 4‐di‐tert‐butylphenol, trans‐2‐octenal, n‐hexadecanoic acid, and nonanoic acid, are observed to be typical aroma compounds in Pu‐erh raw tea, which contribute grass and fat. Interestingly, except these acids, acetic acid, hexanoic acid, octanoic acid, and propanoic acid are also found in Pu‐erh raw tea, but they are not currently identified as aroma‐active compounds (Zhai et al., 2022). For instance, as an endogenous metabolite, 2, 4‐di‐tert‐butylphenol is obtained from the alkylation reaction of phenol and isobutene, so it is generally a major component of volatile oil in many organisms (Yang et al., 2016; Zhao et al., 2020). 2, 4‐di‐tert‐butylphenol owns a higher relative content of total acids even of total volatiles in Pu‐erh raw tea (11.2–17.0%, the highest from MH and the lowest from LX), accounting for 57.6–65.9% of total acids and 12.6–19.0% of total volatiles (Table 2).

### 3.2 The distribution of volatile enantiomers in various origin places

**FIGURE 3:**
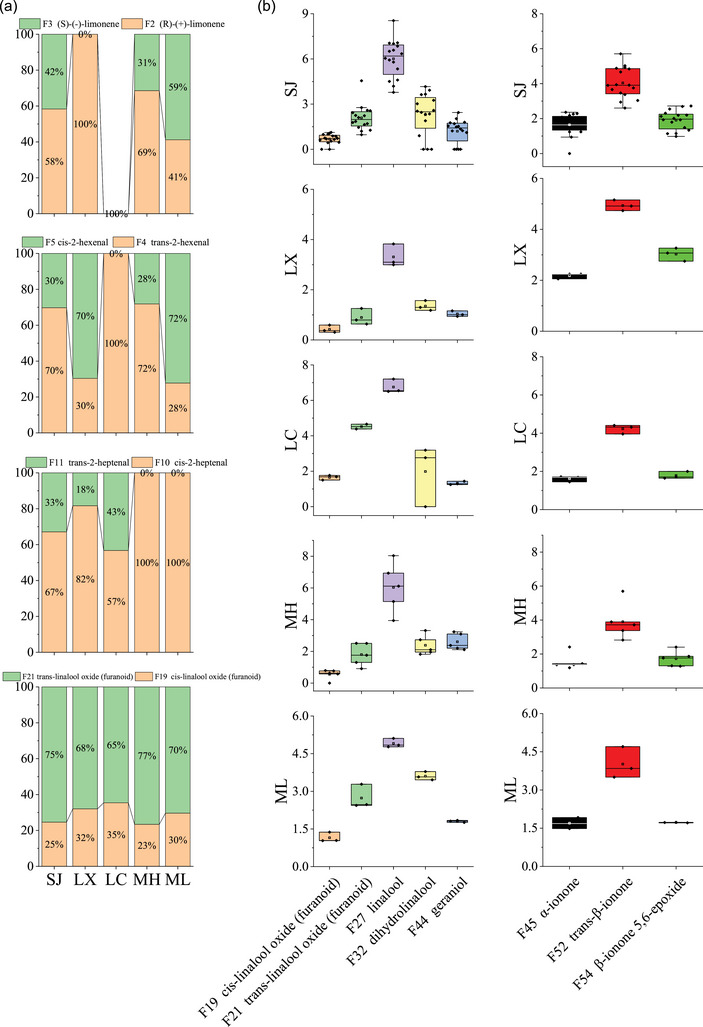
Analysis of volatile enantiomers in five origin places. (a) Distribution of various enantiomers. (b) Metabolites of linalool and ionone.

Enantiomeric analysis of volatile compounds is a prevalent topic in the realm of food chemistry, particularly in the study of tea. It is noteworthy that many volatile compounds in tea possess stereocenters, resulting in enantiomers with distinct aroma profiles and thresholds. In the study, four pairs of volatile enantiomers were detected in this study, including limonene (S‐ and R‐), hexenal (E‐ and Z‐), heptenal (E‐ and Z‐), and linalool oxide (furanoid) (E‐ and Z‐) (Figure 3a). The ratios of some volatile enantiomers in dark teas were obviously different from those in dark raw tea after microbial fermentation. As was mentioned above, the properties of the tea cultivars and the influence of tea processing resulted in both similarity and inconsistency on the essential aromatic components related to the tenderness and maturity of tea shoots among different tea sample series; this was demonstrated by the relevant Venn diagram.

Linalool is a major volatile component of tea aroma with an entirely different sensorial property. Linalool oxide (furanoid) is considered a potential chemical marker in the mature leaves, which will be released from the fixation and drying steps of tea. As shown in Figure 3a, the ratios of trans‐linalool oxide (furanoid) are slightly higher than those of cis‐linalool oxide (furanoid) in these samples. According to a recent study by Zhou, the proportion of enantiomers such as linalool is mainly determined by the enzyme catalytic efficiencies and relative gene expression levels. However, linalool can be bio‐transformed with fungal catalysis in culture media, and the biotransformation of R‐linalool by *Aspergillus niger* is almost trans‐furanoid and trans‐pyranoid linalool oxide (W. Ma et al., 2021). In this study (Figure 3), the enantiomers of linalool oxides A and B are mainly dominated by the (2S,5S)‐type and (2S,5R)‐type enantiomers, respectively. The distributions of (2S,5S)‐linalool oxide A are 65–77% in these five sites (with the highest ratio in MH), while the distributions of (2S,5R)‐linalool oxide B are 23–35% (with the highest ratio in LC). Similar to linalool oxide (furanoid), linalool is sourced from the methylerythritol phosphate (MEP) pathway in the tea plants and is generated from the corresponding glycosidically bound precursors when teas are processed (W. Ma et al., 2021). Geranyl pyrophosphate (GPP), a key precursor of MEP, is used to indirectly biosynthesis geraniol, nerol, linalool, and carotene during the secondary metabolism of the tea plant. In particular, the linalool oxide is formed after oxidation and further hydrolysis under the acidic condition, while β‐carotene generates β‐ionone through lyase, and β‐ionone‐5,6‐epoxide is substituted by an epoxy group across positions 5 and 6 from β‐ionone. In this study, there exists a higher relative content of linalool in five places (3.31–6.75%, highest in LC and lowest in LX, LC > MH, SJ > ML > LX) compared with β‐ionone (3.03–4.23%, highest in LC and lowest in LX, LC > SJ, ML > MH > LX), and between the both the former is sourced from a similar volatile terpenoid pathway and the latter constitutes the carotenoid derived aromatic compounds. Notably, the formation of the tea aroma is deeply affected not only by the absolute contents of volatiles but also by the interaction and coordination of each aromatic component in tea.

S‐limonene exhibits a lemon‐like aroma, whereas R‐limonene exhibits a turpentine smell (Shao et al., 2022). The ratios of R‐limonene (41–69%) are slightly higher than those of S‐limonene (31%‐59%) in SJ, MH, and ML, except for LX which had a 100 ratio of R, while it has not been detected in LC. Interestingly, it has been reported that limonene can be converted into α‐terpineol (0.84–2.21%, highest in MH and lowest in LX in this study) by *Penicillium digitatum*, and R‐limonene is converted into R‐α‐terpineol more easily than S‐limonene (W. Ma et al., 2021). Trans‐2‐heptenal exhibits a fruity aroma, whereas cis‐2‐heptenal exhibits a mushroom‐like smell. In contrast, the ratios of cis‐2‐heptenal (57–82%, highest in LX) are much higher than trans‐2‐heptenal (18–43%, highest in LC), and there is only cis‐2‐heptenal detected in MH and ML particularly. Trans‐2‐hexenal exhibits a fruity and floral aroma, whereas cis‐2‐hexenal exhibits a fatty‐like smell. The ratio of trans‐2‐hexenal (70–72%) and cis‐2‐hexenal (28%‐30%) in SJ and ML is the same as between cis‐2‐hexenal (70–72%) and trans‐2‐hexenal (28–30%) in LX and ML, both of which equals to two times, however, there is only trans‐2‐hexenal detected in LC. Previous studies have shown that the distribution of volatile enantiomers in tea is mainly related to the variety and processing of tea and the origin environment. In particular, the different conversion or generating rates might be responsible for the differences between the two enantiomers of each volatile compound (such as limonene, 2‐hexenal, 2‐heptenal, and linalool oxides). The distribution of volatile enantiomers can be also greatly influenced by microbial fermentation. Enantioselective catalysis by microbial enzymes in postfermentation of Pu‐erh raw tea may lead to ratio changes in these volatile enantiomers (Shao et al., 2022; Figure 3).

### 3.3 Multivariate analysis of Pu‐erh raw tea

**FIGURE 4:**
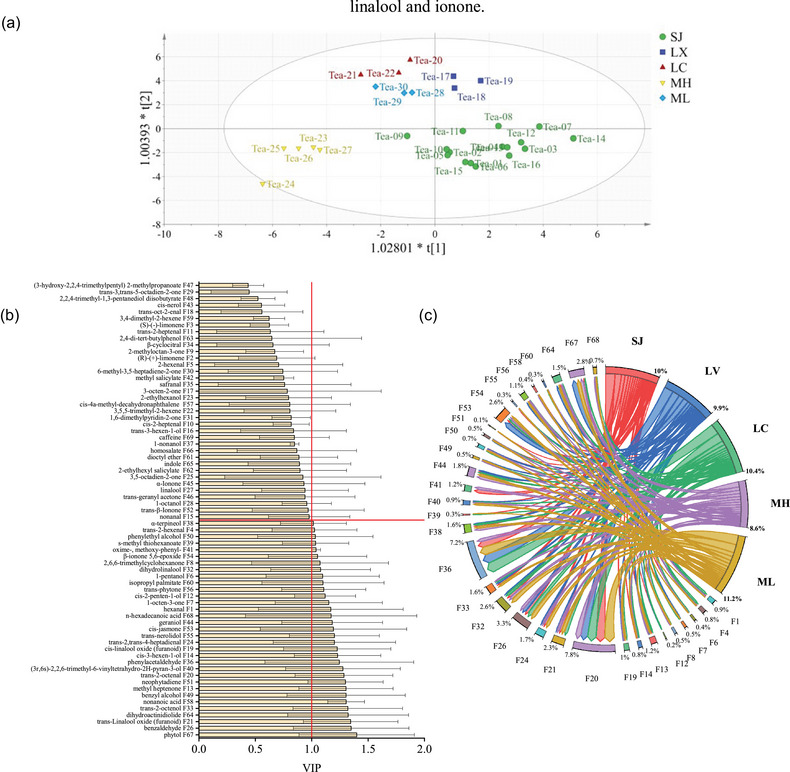
OPLS‐DA analyses of raw tea samples from different regions. (a) OPLS‐DA scores. (b) Volatile component index VIP chart. (c) Chord diagrams of VIP > 1 and *p* < 0.05 (Holm–Bonferroni). In chord diagram, lines indicate the correspondence between locations and volatiles, and the line width indicates the relative contents of volatiles.

To gain insight into the differences between the volatile components of Pu‐erh raw tea, the OPLS‐DA method is applied for multivariate statistical analysis. As illustrated in Figure 4a, satisfactory discrimination is obtained based on the volatile contents, since the model parameters (*R*(*x* ^2^) = 0.926, *R*(*y* ^2^) = 0.843, and *Q* ^2 ^= 0.501) demonstrated the high dependability of this model (Yun, 2020). It is distinct to distinguish the origin place of SJ and MH through their volatile contents, because of the gathering effect of the two. Two‐place limits of LX, LC, and ML are not very obvious; however, they can differentiate from each other. Variable importance in the projection (VIP) is performed to screen out the significant volatile components contributing to discriminating the five groups. The judging criteria with VIP > 1.0 are generally considered to play an important role in aroma quality, where 33 volatile compounds are screened out in total (Figure 4b). The variations in the concentrations of these compounds provide a more comprehensive understanding of the background, richness, and roundness of the overall aroma (P. Liu et al., 2022). They contribute floral, fruity, grass, honey, fatty, woody, and nutty fragrances that bring pleasure to tea drinkers. These volatiles may be the characteristic compounds that distinguish Pu‐erh raw tea from five origin places. The chord diagram is a flow diagram, indicating the direction from nodes (origin places) to each volatile in Pu‐erh raw tea. The relative contents of 33 volatiles are in the following order: ML (50.6%) > LC (48.5%) > LX (44.9%) > SJ (44.3%) > MH (39.1%) (Figure 4c). For instance, trans‐2‐octenal (F20), phenylacetaldehyde (F36), benzaldehyde (F26), phytol (F67), trans‐linalool oxide (furanoid) (F21), dihydro‐linalool (F32) and β‐ionone 5,6‐epoxide (F54) show a higher content and kindly appear in the major aroma in Pu‐erh raw tea.

### 3.4 KEGG pathway analysis of volatiles of Pu‐erh raw tea

**FIGURE 5:**
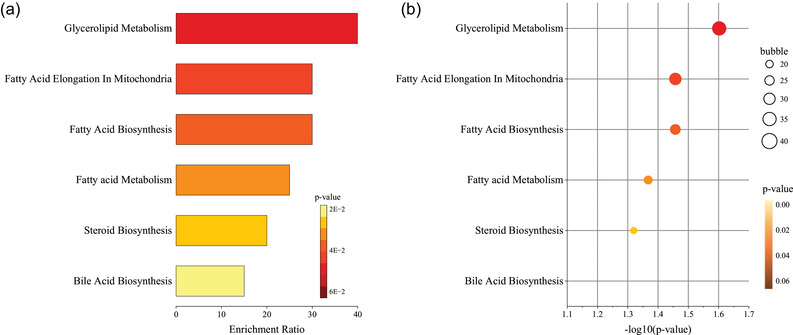
Barplot and dotplot based on 33 characteristic compounds.

According to the top 50 characteristics with VIP > 1 and *p* < 0.05 (Holm–Bonferroni), a KEGG pathway analysis is performed. As shown in Figure 5, the enrichment pathways of the characteristic compounds are mainly concentrated in the mevalonate pathway, MEP/DOXP pathway, steroid biosynthesis, fatty acid biosynthesis, glycerolipid metabolism, bile acid biosynthesis, etc., among which the glycerolipid metabolism and fatty acid biosynthesis are more enriched. On the other hand, this also proved that the metabolic processes of glycerolipid can be better regulated by Pu‐erh raw tea, which is consistent with the biological health effects reported in the literature (Gou et al., 2023; Lv et al., 2014), which can be used as a reference for future research on postfermentation and active substance accumulation in Pu‐erh raw tea.

**FIGURE 6:**
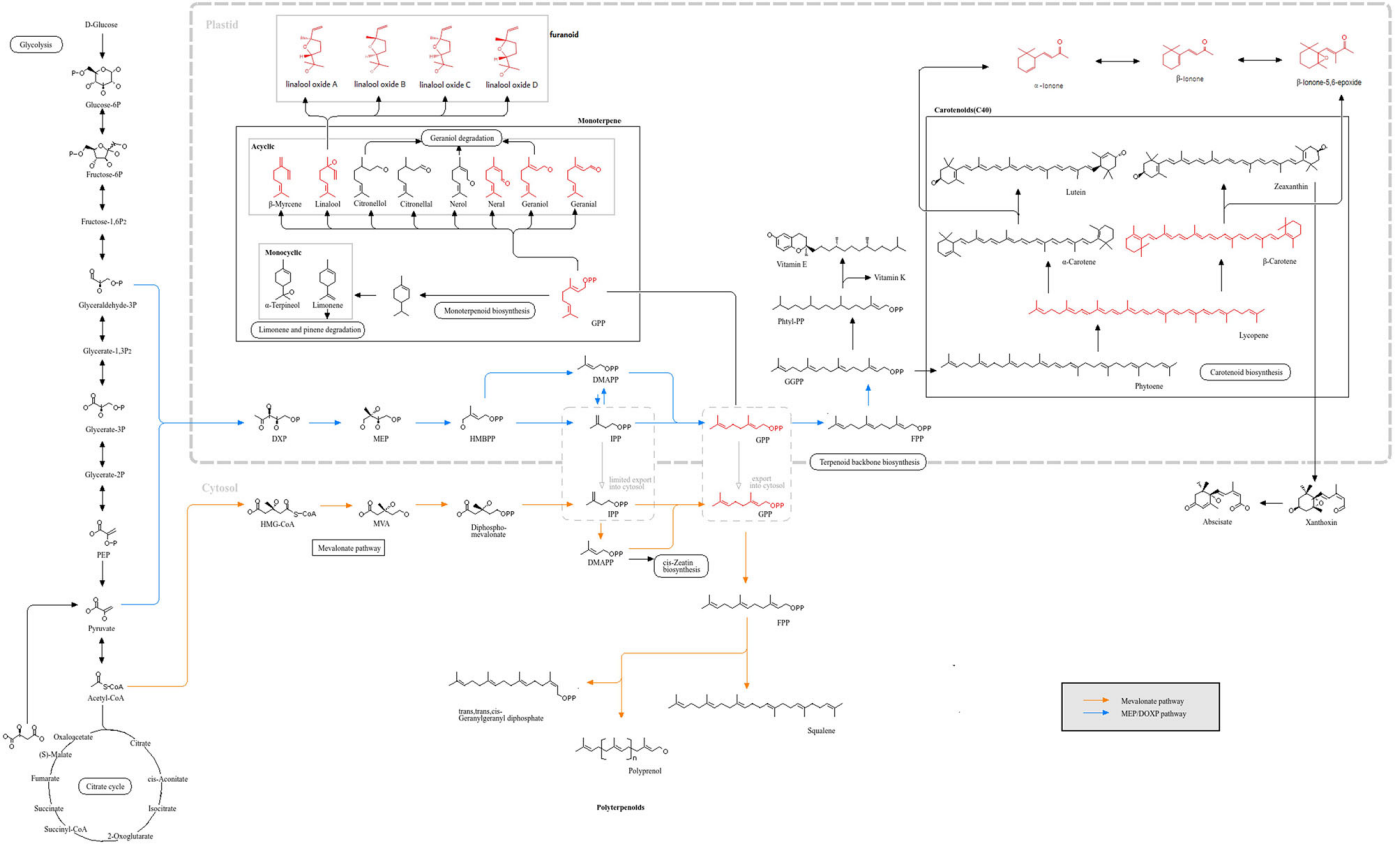
Enrichment pathway of KEGG. *Source*: Shao et al. (2022); W. Ma et al. (2021); C. Wang et al. (2016); KEGG: Kyoto Encyclopedia of Genes and Genomes.

Postfermented Pu‐erh raw tea is produced by spontaneous fermentation under high humidity, during which processing several metabolisms will be promoted or suppressed. First, the “green,” “pleasant,” and “fruity” attributes are weakened due to changes in the levels of hexanal and (E)‐2‐hexenal. Engaged in the biosynthetic pathway of steroids and the metabolic pathway of fatty acids, it serves as the primary decomposition product of linoleic acid oxidation, a process associated with the generation of undesirable flavors. There are five production regions characterized by relatively high levels of LC and low levels of ML. Second, the linalool oxide (furanoid) in the fresh tea leaves, the well‐known biosynthesis pathway of volatile terpenoids during the secondary metabolism of the tea plant, may be indirectly biosynthesized by the plastidic MEP pathway with GPP (the precursors of linalool) under the acidic condition. It will be released from the precursors by enzymatic hydrolysis or thermolysis during the drying steps showing diverse changes in five places because of various processing in each plant, with higher relative content in LC and lower content in LX. Third, as the indirect precursors of methyl salicylate, benzaldehyde could be directly generated from the methylation of salicylic acid by the catalysis of salicylic acid carboxyl methyltransferase. There are five production regions characterized by relatively high levels of ML and low levels of MH. Fourth, phenylacetaldehyde produced from phenylalanine via Strecker degradation is a key odorant responsible for the sweet aroma. Strecker degradation typically occurs during high‐temperature fixation and drying processes. In five production areas, LC significantly increased the levels of Strecker degradation products (such as benzaldehyde) compared with MH, resulting in a sweet and mellow aroma. Fifth, as key odorants of the sweet and floral aromas, linalool and geraniol are derived from the hydrolytic release of glucosides and primeverosides—a way that enzymes released from injured tea tissues into cell walls hydrolyze glycosidic bonds and release volatile aromas. Under specific temperature and humidity conditions, the nonenzymatic hydrolysis of glycoside precursors facilitates the release and subsequent accumulation of linalool and geraniol. This process enhances the floral and sweet aromatic profile of ML, whereas LX exhibits a comparatively lighter aroma. Sixth, β‐ionone derived from carotenoid degradation is one of the key floral odorants, which is mainly affected by temperature and moisture. When the temperature is 25–45°C, it could promote the degradation of β‐carotene, and the hot and humid environment can provide the necessary reaction conditions. The humid and hot environment of LX can provide the necessary reaction conditions for the degradation of β‐carotene, promoting the production of β‐ionone, while ML is lower. All in all, due to the different growth environments, planting modes, management methods, and processing ways in diverse production areas, there are significant diversities in the production or degradation of the main aroma components in Pu‐erh raw tea, forming unique flavors and qualities of Pu‐erh raw tea in varying regions (Figure 6; G. Liu et al., 2024; P. Liu et al., 2022; Y. Ma et al., 2021; Shao et al., 2022; Wei et al., 2024).

## 4 CONCLUSION

In the present study, a method combining HS‐SPME with GC‐MS was used to profile the volatile compounds in Pu‐erh raw tea from five different origins (SJ, LX, LC, MH, ML). In total, 69 volatile compounds including alcohols, aldehydes, ketones, esters, acids, terpenes, alkenes, and others were identified, and alcohols, aldehydes, and ketones were the top three classes of volatiles. Moreover, four pairs of volatile enantiomers were detected, containing limonene (S‐ and R‐), hexenal (E‐ and Z‐), heptenal (E‐ and Z‐), and linalool oxide (furanoid) (E‐ and Z‐), and their ER values appeared in a significant variety in 5 places. Then, through the OPLS‐DA method, it was revealed that satisfactory discrimination is obtained based on the volatile contents. The judging criteria with VIP > 1.0 were generally considered to play an important role in aroma quality, where 33 volatile compounds were screened out in total. Ultimately, according to the top 50 characteristics with VIP > 1 and *p* < 0.05 (Holm–Bonferroni), a KEGG pathway analysis was performed, which was mainly concentrated in the mevalonate pathway, MEP/DOXP pathway, steroid biosynthesis, fatty acid biosynthesis, glycerolipid metabolism, bile acid biosynthesis, etc. The diverse traditional postfermentation techniques have had a significant impact on the volatile profiles of different varieties of Pu‐erh raw tea. While extensive research has been conducted to characterize the key flavor components of Pu‐erh raw tea, there remain numerous unresolved inquiries that are crucial for future investigation, enhancing our comprehension and scientific assessment of tea aroma, and laying a foundational theoretical framework for discerning tea origins. For example, further studies should concentrate on compounds that are absent in fresh tea leaves but develop during the fermentation process (i.e., fermentation and drying) from precursor molecules lacking in odor or taste. Additionally, emphasis should be placed on investigating the flavor distinctions in Pu‐erh raw tea derived from various plant cultivars, geographical locations, and production techniques. The answers to these questions will make possible the evaluation of sensory attributes of tea products at a molecular level.

## AUTHOR CONTRIBUTIONS

**Lijuan Du**: Writing—original draft; writing—review and editing. **Yanping Ye**: Software; data curation. **Jinliang Shao**: Investigation. **Yutong Wang**: Methodology; formal analysis. **Guolei Zhu**: Resources. **Hua Jiang**: Resources. **Hongcheng Liu**: Funding acquisition. **Zhenhuan Liu**: Funding acquisition.

## CONFLICT OF INTEREST STATEMENT

The authors declare no conflict of interest.
